# REIIBP methylates nucleolar proteins and regulates pre-rRNA processing

**DOI:** 10.1016/j.jbc.2025.110609

**Published:** 2025-08-16

**Authors:** Qianqian Yang, Xiaochun Yu

**Affiliations:** 1 School of Life Sciences, Fudan University, Shanghai, China; 2 Westlake Laboratory of Life Sciences and Biomedicine, Hangzhou, Zhejiang, China; 3 School of Life Sciences, Westlake University, Hangzhou, Zhejiang, China; 4 Institute of Basic Medical Sciences, Westlake Institute for Advanced Study, Hangzhou, Zhejiang Province, China

**Keywords:** pre-rRNA, protein methylation, REIIBP, nucleolus

## Abstract

The *NSD2* (nuclear receptor binding SET [Suppressor of variegation 3–9, Enhancer of zeste, and Trithorax] domain protein 2) gene encodes a short isoform, REIIBP (interleukin-5 response element II binding protein), *via* alternative transcription initiation. It is particularly overexpressed in t(4;14)+ multiple myeloma. However, the biological functions of REIIBP remain elusive. Here, we show that REIIBP localizes at the dense fibrillar component of the nucleolus. The disordered regions of REIIBP recognize pre-rRNA, which mediates the nucleolar localization of REIIBP. Proteomic analyses reveal that REIIBP is associated with many pre-rRNA processing factors and mediates lysine methylation on a set of pre-rRNA processing factors. Moreover, REIIBP dysregulates pre-rRNA processing, mediated by both disordered regions and the SET domain, and ultimately affects ribosome biogenesis. Collectively, these findings uncover the role of REIIBP in the regulation of ribosome biogenesis, which may drive the pathological process in multiple myeloma.

The *NSD2* (nuclear receptor binding SET [Suppressor of variegation 3–9, Enhancer of zeste, and Trithorax] domain protein 2) gene localizes on chromosome 4p16.3 and can produce two protein isoforms, namely full-length MMSET (multiple myeloma [MM] SET domain) and a truncated version, REIIBP (interleukin-5 response element II binding protein), *via* alternative transcription initiation events (1, 2, 3). REIIBP originates independently within the ninth intron of the *MMSET* gene and initiates translation at exon 15 (3). Its sequence and functional domains correspond to the C terminus of MMSET, spanning 584 amino acids (1, 2, 3).

The full-length MMSET protein is a multidomain protein containing a catalytic SET domain, two PWWP (proline–tryptophan–tryptophan–proline motif) domains, a High Mobility Group box, and five PHD (plant homeodomain) zinc finger motifs (4). In contrast, REIIBP retains the entire SET domain, a C-terminal PWWP domain, and two PHD domains (4). Among these modules, the MMSET N-terminal PWWP domain has been shown to recognize nucleosomes with H3K36me2 through a conserved aromatic cage (5). Recently, a chemical probe, UNC6934, has been developed to selectively bind to the aromatic cage of the MMSET N-terminal PWWP domain, thereby disrupting its interaction with H3K36me2 nucleosomes (6). Interestingly, targeting the N-terminal PWWP domain of MMSET with UNC6934 alters the subcellular localization of MMSET, leading to its accumulation in the nucleolus (6). Currently, MMSET is considered a histone methyltransferase, specific for H3K36me2 (7, 8, 9). Studies have shown that MMSET overexpression leads to a global increase in H3K36me2 levels and a concurrent decrease in H3K27me3 levels (10, 11). Furthermore, knockdown of MMSET in human cells results in decreased proliferation, partly because of the loss of H3K36me2 (8, 9). In mice, MMSET deficiency leads to perinatal mortality, growth retardation, and craniofacial defects reminiscent of those seen in Wolf–Hirschhorn syndrome (12, 13).

Despite the extensive research on MMSET, studies on REIIBP remain limited. REIIBP is drastically expressed in t(4;14)+ MM (3). The t(4;14) translocation, involving the *IgH* (immunoglobulin heavy chain) locus on chromosome 14q32.33 and the *NSD2* gene on chromosome 4p16.3, occurs in 15% to 20% of MM cases and is associated with a significantly worse prognosis compared with other genetic subgroups (14, 15, 16). The t(4;14) translocation leads to the overexpression of REIIBP, which is driven by the enhancer of the *IgH* gene (3). Although REIIBP retains the C-terminal methyltransferase domain, it is unclear if REIIBP is still able to methylate nucleosomal histones. In addition, other studies suggest that REIIBP may bind to the survival of motor neuron complex, modulating the cellular abundance of snRNPs and subsequently affecting mRNA splicing (17). However, the biological functions of REIIBP and its pathogenic mechanism in MM remain poorly understood and require further investigation.

While both MMSET and REIIBP have been implicated in the pathogenesis of t(4;14)+ MM, only REIIBP is localized in the nucleolus (3). In contrast, MMSET is primarily found in the nucleoplasm outside the nucleolus (3). This distinct subcellular localization suggests that MMSET and REIIBP may play divergent roles in MM pathogenesis through different mechanisms. Since pre-rRNA is transcribed and processed in the nucleolus, the unique subcellular localization of REIIBP indicates that it may participate in ribosome biogenesis.

Interestingly, recent studies have revealed the importance of ribosome biogenesis in MM development (18). Ribosome biogenesis encompasses the synthesis and processing of rRNA, the assembly of rRNA and ribosomal proteins (RPs), and the transport of preribosomal subunits into the cytoplasm, where they form functional ribosomes for mRNA translation (19, 20, 21, 22). Most of these processes occur within the nucleolus. In a recent clinical trial, Kylee *et al*. (23)evaluated the selective RNA polymerase I inhibitor, CX5461, which suppressed pre-rRNA transcription, and observed preliminary antitumor activity in MM patients.

In this study, we demonstrate that REIIBP methylates a set of pre-rRNA processing factors and dysregulates pre-rRNA processing, which may reveal a molecular mechanism of MM development.

## Results

### REIIBP localizes in the dense fibrillar component of the nucleolus

**Figure 1:**
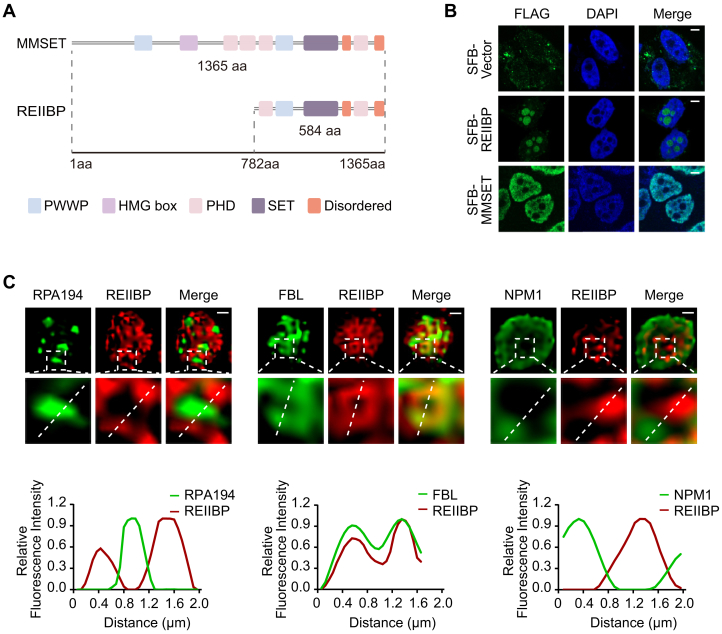
**REIIBP localizes in the DFC of the nucleolus**. *A*, schematic diagram of MMSET and REIIBP. *B*, REIIBP does not colocalize with MMSET. SFB-tagged MMSET or REIIBP were stably expressed in HeLa cells and examined by immunofluorescence (IF) with anti-FLAG antibody. The nucleus and nucleoli were indicated by DAPI staining. Image bar represents 5 μm. *C*, REIIBP exists in DFC. REIIBP (*red*) and indicated nucleolar markers (*green*) were examined by structured illumination microscopy (SIM) in HeLa cells. RPA194, FBL, and NPM1 were used as markers for FC, DFC, and GC, respectively. The fluorescence intensity on the *white dashed lines* was plotted (*lower panels*). Scale bar represents 1 μm. DAPI, 4′,6-diamidino-2-phenylindole; DFC, dense fibrillar component; FC, fibrillar center; GC, granular component; HMG box, high mobility group box domain; MMSET, multiple myeloma SET domain; PHD, plant homeodomain zinc finger; PWWP, named for a conserved Pro–Trp–Trp–Pro motif; REIIBP, interleukin-5 response element II binding protein; SET, Suppressor of variegation 3–9, Enhancer of zeste, and Trithorax.

To mimic the overexpression of REIIBP in the t(4;14)+ MM and study its biological functions, REIIBP was ectopically expressed in HeLa cells (Fig. S1*A*). Of note, the endogenous REIIBP is undetectable in HeLa cells (17). Since REIIBP contains a complete SET domain (Fig. 1*A*), we investigated whether REIIBP also exhibits H3K36 methylation activity similar to MMSET. However, Western blotting assays show that ectopically expressed REIIBP did not change the global levels of H3K36me2 (Fig. S1*B*).

Next, we performed immunofluorescence (IF) staining to examine the subcellular localization of REIIBP. The results reveal that REIIBP and MMSET exhibit mutually exclusive subcellular distributions within the nucleus (Fig. 1*B*). Specifically, REIIBP was localized in the nucleolus, whereas MMSET was found in the nucleoplasm, excluded from the nucleolus (Fig. 1*B*). Consistent with this observation, endogenous REIIBP in the MM cell line NCI-H929 also localizes in the nucleolus (Fig. S1*C*). These completely distinct localization patterns suggest that REIIBP and MMSET perform different functions.

The human nucleolus is composed of 3 phase-separated regions termed fibrillar center (FC), dense fibrillar component (DFC), and granular component (GC) (24). Pre-rRNA transcription occurs at the boundary between the FC and DFC, whereas pre-rRNA processing and modification primarily take place in the DFC, and maturation of preribosomal particles occurs in the GC (24, 25). We utilized structured illumination microscopy (SIM) to further determine the specific subnucleolar (NO) localization of REIIBP, with RPA194, fibrillarin (FBL), and NPM1 as markers for the FC, DFC, and GC, respectively. Our results show that REIIBP colocalized with FBL but not RPA194 or NPM1, indicating its primary localization in the DFC of the nucleolus (Fig. 1*C*).

### The disordered regions target REIIBP to nucleolus

**Figure 2:**
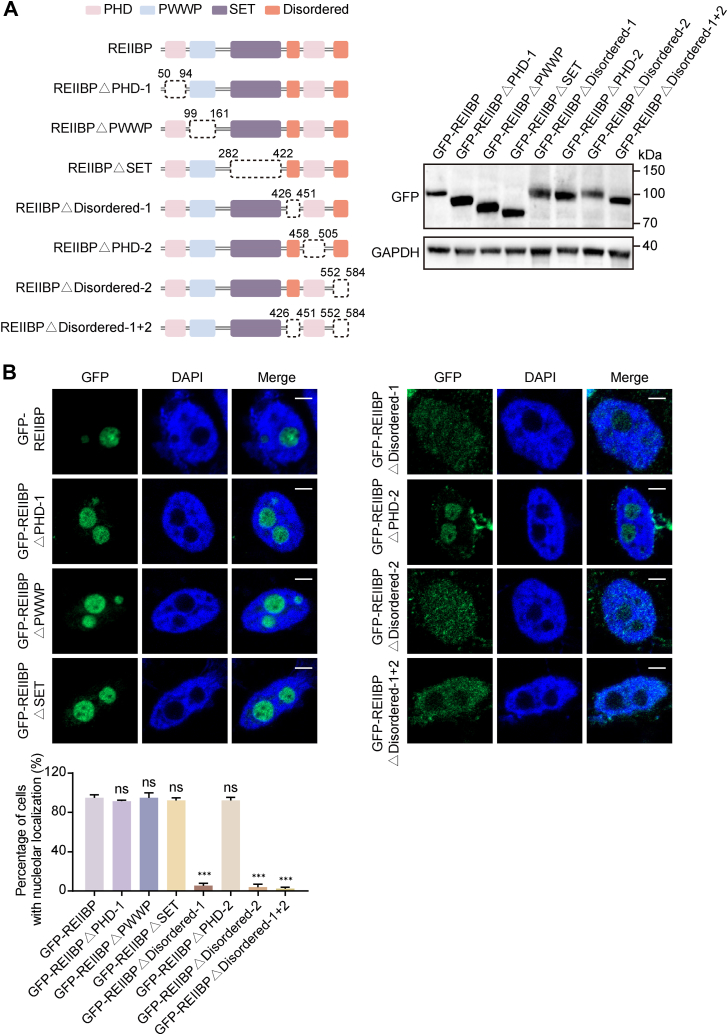
**The disordered regions of REIIBP determine the nucleolar localization of REIIBP**. *A*, schematic for REIIBP domain–deletion mutants (*left panel*). The expression of full-length REIIBP and its domain-deletion mutants in HeLa cells was examined by WB assay (*right panel*). *B*, deletion of either disordered regions abolishes the nucleolar localization of REIIBP. The GFP-tagged full-length REIIBP and its domain-deletion mutants were expressed in HeLa cells and examined by IF. Image bar represents 5 μm. The percentage of the cells with positive GFP signal in nucleolus was calculated (n = 150) (*lower panel*). ∗∗∗*p* < 0.001, ns, not significant. IF, immunofluorescence; REIIBP, interleukin-5 response element II binding protein; WB, Western blotting.

REIIBP contains several functional domains, including an entire SET domain, a C-terminal PWWP domain, and two PHD domains (Fig. 1*A*). To further investigate which domain is required for the NO localization of REIIBP, we generated truncated mutants of REIIBP by deleting each domain. These mutants were expressed in HeLa cells (Fig. 2*A*). The IF staining reveals that deletion of either disordered region, but not other modules, resulted in the abrogation of NO localization of REIIBP (Fig. 2*B*). Thus, these results suggest that these two disordered regions may recognize signals in the nucleolus.

### REIIBP interacts with pre-rRNA

**Figure 3:**
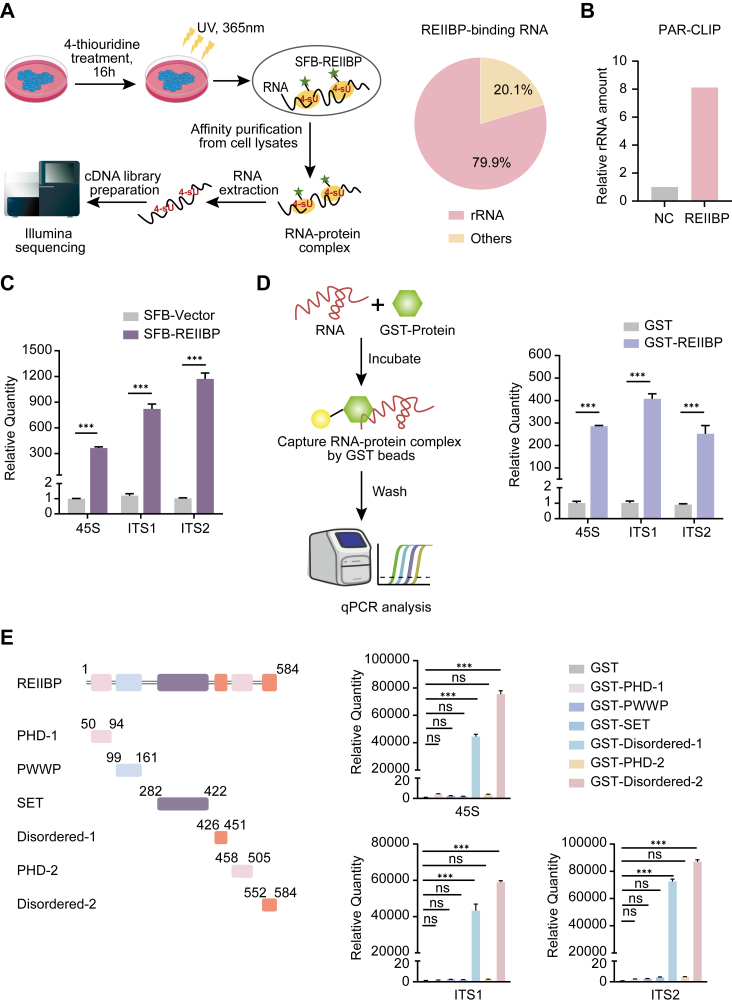
**REIIBP interacts with pre**-**rRNA**. *A*, the procedure of PAR-CLIP and RNA-Seq using HeLa cells stably expressing SFB-REIIBP (*left panel*). The RNA species isolated from the cells are shown (*right panel*). *B*, the rRNA enriched from HeLa cells stably expressing SFB-REIIBP is compared with that from HeLa cells stably expressing SFB-vector alone. The relative fold of rRNA enrichment is calculated as the percentage of rRNA reads among the reads on all peaks detected by RNA-Seq times the absolute amount of total RNA purified in the PAR-CLIP assays. *C*, validation of the interaction between REIIBP and pre-rRNA. Following PAR-CLIP assay, RT–qPCR was performed to examine REIIBP-bound pre-rRNAs. ∗∗∗*p* < 0.001. *D*, diagrammatic drawing of *in vitro* GST pull-down-qPCR assay (*left panel*). Recombinant REIIBP protein binds pre-rRNA *in vitro* (*right panel*). GST or GST-REIIBP proteins were incubated with total RNA extracted from HeLa cells. RT–qPCR was performed to examine REIIBP-associated pre-rRNAs using primers targeted on 5′ETS, ITS1, and ITS2 regions of pre-rRNA. ∗∗∗*p* < 0.001. *E*, schematics of the REIIBP protein domain structure and its truncated mutants are shown (*left panel*). The disordered regions of REIIBP bind to pre-rRNA (*right panel*). Recombinant truncated REIIBP variants were incubated with total RNA extracted from HeLa cells. *In vitro* GST pull-down-qPCR was performed to determine the interaction between truncated REIIBP variants and pre-rRNA. ∗∗∗*p* < 0.001, ns, not significant. 5′ETS, 5′ external transcribed spacer; GST, glutathione-S-transferase; ITS, internal transcribed spacer; PAR-CLIP, photoactivatable ribonucleoside–enhanced crosslinking and immunoprecipitation; qPCR, quantitative PCR; REIIBP, interleukin-5 response element II binding protein.

Accumulated evidence suggests that disordered regions often recognize RNA species (26, 27, 28). Thus, we ask if these two disordered regions of REIIBP may also associate with RNA species. We used 4-thiouridine to label newly synthesized RNA and performed photoactivatable ribonucleoside–enhanced crosslinking and immunoprecipitation (IP) (PAR-CLIP) assay combined with RNA-Seq (Figs. 3*A* and S2*A*). Interestingly, we found that the predominant RNA species associated with REIIBP was rRNA (Fig. 3*A*). Since rRNA is 80% to 90% of total RNA (29, 30), it is unlikely to observe significant changes in the percentage of rRNA in overall purified RNA species. However, the amount of purified rRNA from PAR-CLIP was remarkably increased (Fig. 3*B*), indicating that the rRNA identified in the PAR-CLIP-Seq assays was not common RNA contamination but specifically associated with REIIBP.

Given that REIIBP is localized in the nucleolus, where pre-rRNA is transcribed and processed, we designed primers targeting the 5′ external transcribed spacer (5′ETS) and two internal transcribed spacer (ITS1, ITS2) regions of pre-rRNA and performed PAR-CLIP–quantitative PCR (qPCR) to further validate the results from the PAR-CLIP-Seq assays. Again, we found that REIIBP was associated with pre-rRNA (Fig. 3*C*). We also examined other abundant noncoding RNAs, such as 7SK and MALAT1, and did not find that REIIBP can interact with these RNA species (Fig. S2*B*). Next, to further determine the RNA-binding specificity, we purified glutathione-*S*-transferase (GST)-REIIBP recombinant protein and performed *in vitro* pull-down assay followed by RT–qPCR (Figs. 3*D* and S2*C*). The results confirm that REIIBP primarily binds pre-rRNA but not other RNA species (Figs. 3*D* and S2*D*). Moreover, we performed an *in vitro* pull-down assay using native pre-rRNA or heat-denatured pre-rRNA and found that heat denaturing largely disrupted the interactions (Fig. S2*E*), suggesting that the pre-rRNA secondary structure may play an important role in mediating the interactions.

To examine if the disordered regions of REIIBP interact with pre-rRNA, we purified recombinant truncated mutants of REIIBP and performed an *in vitro* pull-down-qPCR assay. We found that the two disordered regions at the C terminus of REIIBP but not the other domains interacted with pre-rRNA (Figs. 3*E* and S2*C*). Taken together, these findings indicate that REIIBP selectively interacts with pre-rRNA *via* its disordered regions.

### REIIBP associates with pre-rRNA processing factors

**Figure 4:**
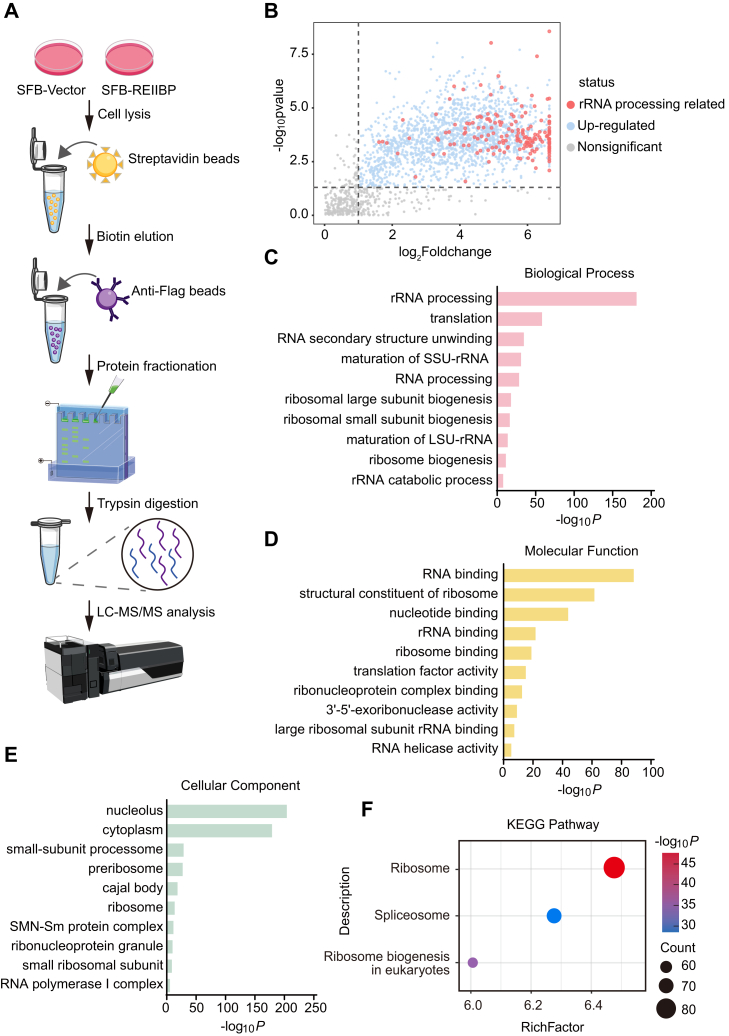
**REIIBP associates with rRNA processing–related proteins**. *A*, schematic diagram of immunoprecipitation (IP)–MS experimental process. *B*, a volcano plot of REIIBP-associated proteins identified by IP–MS. Proteins with significant upregulation (log_2_FC ≥1 and *p* < 0.05) are highlighted in *light blue*, and rRNA processing–related proteins are marked in *light red*. Proteins without significant changes (log_2_FC <1 and *p* ≥ 0.05) are shown in *gray*. The *dashed lines* indicate the thresholds for statistical significance and FC. *C*–*E*, the REIIBP-associated proteins are classified by biological process (*C*), molecular function (*D*), and cellular component (*E*). *F*, the KEGG pathway analysis of the REIIBP-associated proteins. FC, fold change; IP, immunoprecipitation; KEGG, Kyoto Encyclopedia of Genes and Genomes; MS, mass spectrometry; REIIBP, interleukin-5 response element II binding protein.

To further investigate the potential functions of REIIBP, we identified the interacting proteins of REIIBP through proteomics analysis. In HeLa cells stably expressing SFB-REIIBP, two rounds of affinity purification were performed to enrich SFB-REIIBP using streptavidin beads and anti-FLAG beads, followed by mass spectrometry (MS) analysis (Fig. 4*A* and Table S1). The volcano plot highlights the proteins that interact with REIIBP, with the largest and most significantly enriched group being those involved in pre-rRNA processing, which are indicated by light red dots (Fig. 4*B*). Further Gene Ontology analyses reveal that REIIBP-interacting proteins were primarily involved in pre-rRNA processing and ribosome biogenesis (Fig. 4, *C–E* and Table S2). In addition, the Kyoto Encyclopedia of Genes and Genomes pathway analysis also shows similar results (Fig. 4*F* and Table S2).

### REIIBP methylates pre-rRNA processing factors

Since REIIBP contains a complete SET domain and is associated with pre-rRNA processing factors in the nucleolus, we asked if REIIBP may methylate pre-rRNA processing factors in the nucleolus. We employed the subcellular fractionation approach to obtain NO fractions. This approach increases the relative abundance of methylated proteins within this compartment. LC–MS/MS analyses reveal that the methylation levels at 249 lysine sites of 141 proteins were upregulated in HeLa cells expressing REIIBP (Table S3). To validate the results, we examined the MS/MS spectra of peptides with elevated lysine methylation levels. Four representative spectra are presented, with the *m/z* values of observed fragment ions (y- and b-ions) matching the theoretical fragmentation patterns of methylated peptides, thereby confirming the actual presence of methylation modifications on the targeted peptides (Fig. S3*A*). We further analyzed the physical interactions between these candidate substrates and identified the largest network consisting of 43 ribosome biogenesis–associated proteins, most of which are involved in pre-rRNA biogenesis (Fig. S3*B*). Gene Ontology analysis of the upregulated methylated proteins also reveals their enrichment in ribosome biogenesis and rRNA processing (Fig. S3*C*).

**Figure 5:**
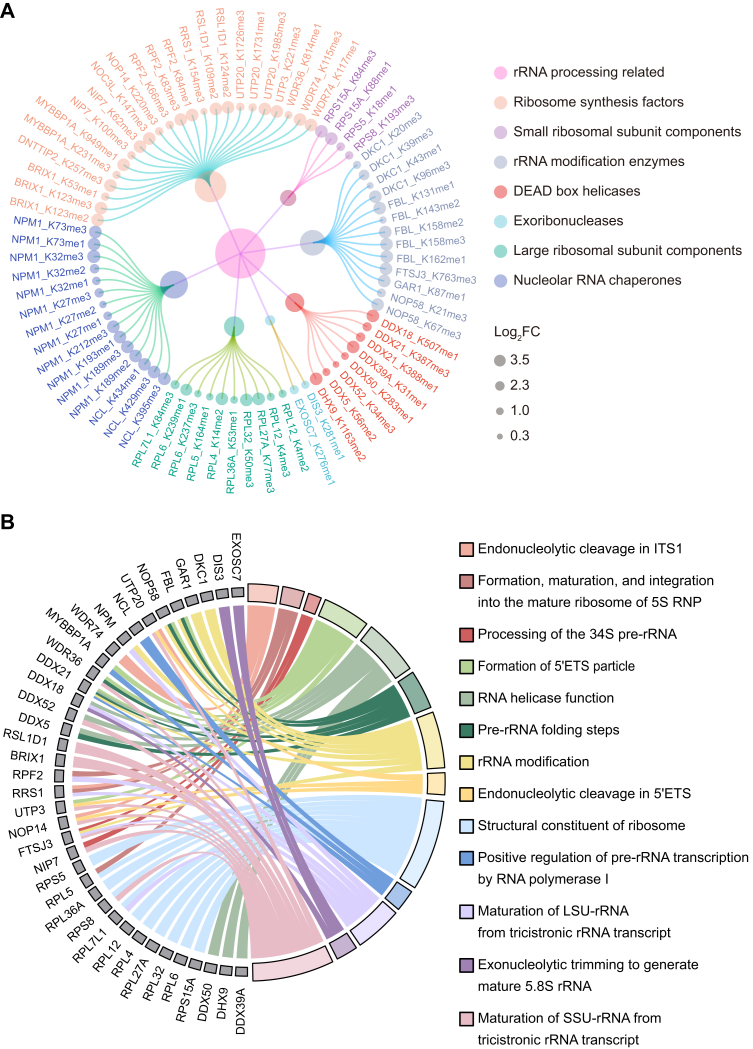
**REIIBP methylates pre-rRNA–processing proteins**. *A*, LC–MS/MS analysis of NO proteins from HeLa cells stably expressing REIIBP reveals increased lysine methylation in rRNA processing proteins. A total of 75 methylated lysine residues (including mono-, di-, and trimethylated forms) across 40 rRNA processing proteins were identified as having elevated levels. These proteins were systematically classified into seven functional categories. The figure visualizes significant methylation changes with fold change >1.3. *B*, the chord diagram displays the specific pre-rRNA processing process involving 38 methylation-upregulated candidate proteins. REIIBP, interleukin-5 response element II binding protein.

Next, we conducted a comprehensive statistical analysis and classification of 40 proteins associated with rRNA processing that exhibited elevated lysine methylation levels. Our findings have identified a total of 75 sites with increased methylation, encompassing monomethylation, dimethylation, and trimethylation of lysine residues. These proteins were categorized into several functional groups, including ribosome synthesis factors, small ribosomal subunit components, rRNA modification enzymes, DEAD box helicases, exoribonucleases, large ribosomal subunit components, and NO RNA chaperones (Fig. 5*A*). The detailed functions of these 40 candidate protein substrates were further reviewed and compiled, with 38 of them reported to be involved in key specific steps of rRNA processing. These steps encompass 13 distinct aspects, including endonucleolytic cleavage in ITS1/5′ETS, rRNA modification, pre-rRNA folding, and processing of the 34S pre-rRNA, among others (Fig. 5*B*) (31, 32, 33, 34, 35, 36, 37).

To further validate the enzymatic activity of REIIBP, we performed *in vitro* methylation assays with EXOSC7 and NIP7, two substrates of REIIBP identified by MS. Indeed, recombinant wild-type REIIBP, but not the catalytically inactive mutant REIIBP (Y311A) (8), methylated EXOSC7 and NIP7 *in vitro* (Fig. S3*D*). Moreover, substitution of K276 of EXOSC7 or K62 of NIP7 to alanine (EXOSC7[K276A], NIP7[K62A]) abolished the methylation events (Fig. S3*E*). Collectively, these results confirm that REIIBP can methylate pre-rRNA processing factors.

### REIIBP regulates pre-rRNA processing

**Figure 6:**
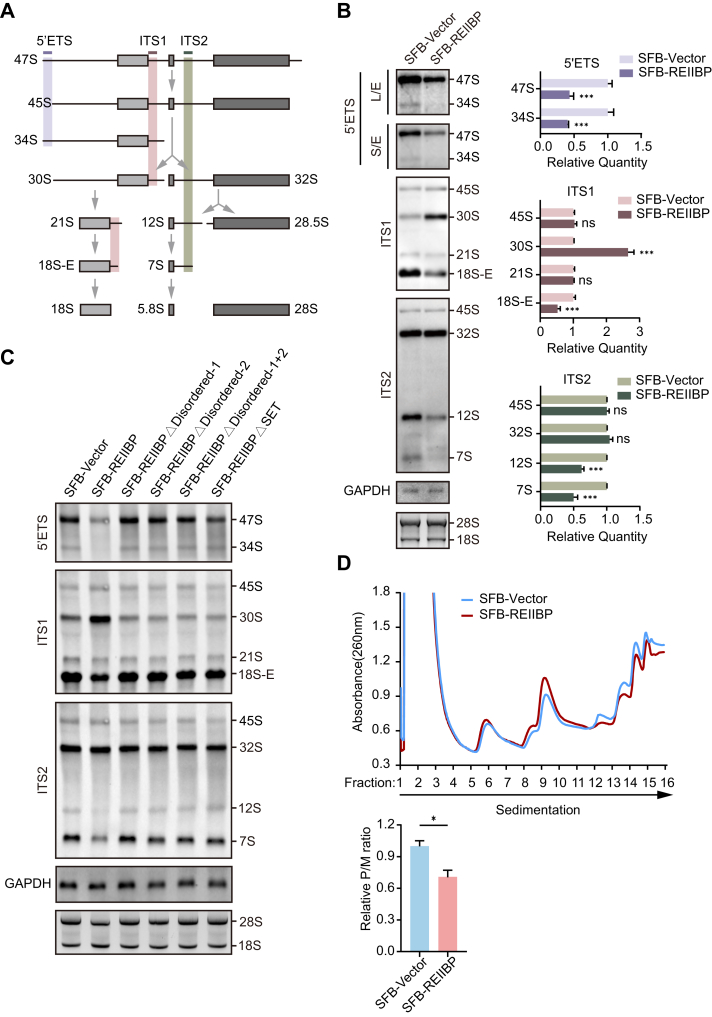
**REIIBP regulates pre-rRNA processing**. *A*, schematic diagram of simplified pre-rRNA processing pathway and the major pre-rRNA intermediates in human cells. The *purple*, *dark red*, and *dark green lines* indicate the location of the 5′ETS, ITS1, and ITS2 hybrid probes, respectively. The *lavender*, *light red*, and *light green* strips cover the pre-rRNA intermediates that the corresponding probe can detect. *B*, REIIBP dysregulates pre-rRNA processing. NB analyses were performed to examine pre-rRNA intermediates in HeLa cells stably expressing SFB-Vector or SFB-REIIBP (*left panel*). Indicated probes are shown. The relative levels of 47S, 45S, 30S, 21S, 18S-E, 32S, 12S, and 7S pre-rRNAs are measured (*right panel*). Values are means ± SD of 3 independent assays. ∗∗∗*p* < 0.001, ns, not significant. *C*, disordered regions and the SET domain of REIIBP are required for the dysregulated pre-rRNA processing. NB analyses were performed with probes targeting the 5′ETS, ITS1, and ITS2. *D*, polysome profiles and polysome-to-monosome (P/M) ratio change upon REIIBP expression. Data are presented as mean ± SD (n = 3); ∗*p* < 0.05. 5′ETS, 5′ external transcribed spacer; ITS, internal transcribed spacer; L/E, long exposure; NB, Northern blotting; REIIBP, interleukin-5 response element II binding protein; S/E, short exposure; SET, Suppressor of variegation 3–9, Enhancer of zeste, and Trithorax.

In human cells, rRNA processing is a multistep and multicircuit process that is both hierarchical and alternative. The human primary pre-rRNA, or 47S pre-rRNA, is transcribed by RNA polymerase I from the approximately 400 head-to-tail tandem repeats of ribosomal DNA located on the short arms of the five acrocentric chromosomes (chr13, 14, 15, 21, and 22) (35). This primary pre-rRNA transcript contains the 18S, 5.8S, and 28S rRNAs, which are flanked by the 5′ETS, ITS1, ITS2, and 3′ETS. Through a series of sequential cleavages by exonucleases and endonucleases at specific sites, the interspaced regions are removed, producing various intermediate products that eventually mature into functional rRNA (Fig. S4). Based on the observation that REIIBP interacts with and methylates a subset of proteins involved in pre-rRNA processing, and considering that post-translational modifications (PTMs) of proteins associated with ribosome biogenesis can influence rRNA biogenesis and ribosome function (38, 39, 40, 41, 42, 43), we hypothesize that REIIBP is involved in the regulation of pre-rRNA processing. Therefore, we designed probes targeting the 5′ETS, ITS1, and ITS2 regions of pre-rRNA and performed Northern blotting (NB) assays to examine the various intermediates produced during pre-rRNA processing (Fig. 6*A*). The results of 5′ETS probe hybridization indicate that expression of REIIBP leads to a reduction of 47S and 34S pre-rRNAs (Fig. 6*B*), which is attributed to enhanced cleavage at the 01 site that occurs prior to the 2 site during the early stages of pre-rRNA processing. Hybridization with the ITS1 probe further reveals that REIIBP expression results in the accumulation of the 30S pre-rRNA intermediates, accompanied by a decrease in the 18S-E pre-rRNA intermediates (Fig. 6*B*). This is caused by increased processing at the 2 site following cleavage at the 01 site, accompanied by inhibition of processing at the A0 site, decreased cleavage at the E site, and enhanced cleavage at the 3 site. Finally, hybridization with the ITS2 probe shows a reduction in both 12S and 7S pre-rRNAs, which can be attributed to the suppression of the cleavage at the 4 site and enhanced exonuclease activity responsible for processing 12S into 7S and subsequently to 5.8S rRNA (Fig. 6*B*). To investigate the physiological role of endogenous REIIBP in pre-rRNA processing, we knocked down *REIIBP* in the MM cell line NCI-H929 with shRNAs (Fig. S5*A*) and performed NB analyses to detect pre-rRNA intermediates. The results demonstrate that REIIBP knockdown increased the levels of 47S, 34S, 21S, 18S-E, 12S, and 7S pre-rRNAs, whereas it decreased the level of 30S pre-rRNA (Fig. S5, *B* and *C*).

Since we have identified that two disordered regions at the C terminus of REIIBP mediate its interaction with pre-rRNA and determine the NO localization of REIIBP (Figs. 2*B* and 3*E*), we further investigated the functional impact of distinct REIIBP domains on pre-rRNA processing. We conducted NB analyses in HeLa cells ectopically expressing full-length REIIBP, the deletion mutants (lacking disordered regions 1/2 or the SET domain), or an empty vector control. Strikingly, expression of mutants lacking either disordered regions 1/2 (individually or combined) or the SET domain shows no detectable effects on pre-rRNA processing intermediates (Figs. 6*C* and S5*D*), indicating that REIIBP-mediated regulation of pre-rRNA processing is dependent on both the disordered regions and the SET domain.

Since rRNA is ultimately assembled into mature ribosomes for protein translation, we investigated the impact of REIIBP on ribosome assembly by polysome profiling. Interestingly, the polysome-to-monosome ratio was clearly reduced in the presence of REIIBP (Fig. 6*D*). Taken together, these results suggest that REIIBP dysregulates ribosome biogenesis.

## Discussion

In this study, we demonstrated that REIIBP localizes to the nucleolus through its interaction with pre-rRNA and plays a critical role in regulating pre-rRNA processing. Functional analyses of REIIBP truncation mutants reveal that this regulatory activity is not solely dependent on the disordered regions, which mediate NO targeting, but also requires the catalytic SET domain. In addition, we identified that REIIBP exhibits methyltransferase activity toward a subset of NO proteins involved in pre-rRNA processing. These findings collectively suggest a dual coordinated role of REIIBP: the disordered regions of REIIBP facilitate its NO localization *via* pre-rRNA binding, where the SET domain subsequently methylates key pre-rRNA processing–associated proteins. This PTM likely modulates the functionality of these target proteins, thereby influencing the efficiency or fidelity of rRNA maturation.

Although our diagram of pre-rRNA processing only shows a sequential and multipathway cleavage of the pre-rRNA to produce mature rRNA (Fig. S4), in fact this process does not occur independently within the cell. It must be synchronized with ribosome assembly (20). The process includes rRNA nucleotide modifications, rRNA folding, and the loading of RPs (20, 21, 35). These highly coordinated processes occur simultaneously and mutually influence each other, involving a large cohort of accessory factors. These include endonucleases and exonucleases, which catalyze the cleavage of pre-rRNA; helicases and chaperone proteins, which participate in rRNA folding; modifying enzymes responsible for rRNA nucleotide modifications; ATPases and GTPases; and numerous RPs that are incorporated as structural and compositional components into the preribosome (19, 36). Therefore, because of the coexistence of multiple pre-rRNA cleavage pathways, the diversity of cooperation among various endonucleases and exonucleases, the crosstalk between accessory factors, and the kinetics and stoichiometric effects of enzymatic reactions, although the direct cause of the changes in intermediate products during pre-rRNA processing observed in our study is the variations in upstream and downstream cleavage efficiencies, the actual underlying cause is likely because of the complex interplay and mutual regulation between the aforementioned processes, which ultimately govern the cleavage efficiencies.

In recent years, accumulated evidence has indicated that ribosome biogenesis is tightly regulated by various PTMs (38, 39, 40, 41, 42, 43). For instance, arginine methylation of human RPS3 and RPS10 is essential for proper ribosome assembly in the nucleolus. Both mutant RPS3 (R64/65/67A) and mutant RPS10 (R158K and R160K), which cannot be methylated, fail to be transported into the nucleolus and are subsequently not incorporated into the ribosome (41, 42). Moreover, RPS6 is involved in the processing of 30S pre-rRNA to 18S rRNA only when its C-terminal five serine residues are phosphorylated (43).

Despite the extensive studies demonstrating that PTMs of NO proteins involved in ribosome biogenesis can regulate rRNA processing and ribosome assembly (38, 39, 40, 41, 42, 43), the potential association of lysine methylation in these processes remains unexplored. Our study demonstrates that (1) REIIBP methylates key pre-rRNA processing factors and (2) REIIBP is essential for pre-rRNA processing, dependent on both its methyltransferase (SET) domain and disordered regions. However, the precise molecular mechanism by which methylation of these factors drives pre-rRNA processing dysregulation remains to be fully elucidated. REIIBP-mediated methylation may potentially impair protein–protein interactions or protein–RNA bindings, as lysine methylation might neutralize positive charges critical for electrostatic partnerships or induce conformational changes through added bulk methyl groups within processing complexes.

In summary, our study demonstrates that REIIBP dysregulates pre-rRNA processing. Additional studies are needed to demonstrate if REIIBP-mediated ribosome biogenesis defects directly promote MM development.

## Experimental procedures

### Antibodies

Antibodies used in this study were purchased from the respective companies: anti-FLAG (Sigma; F1804), anti-REIIBP (Cell Signaling Technology; 65127S), anti-MMSET (Active Motif; 39880), anti-RPA194 (Santa Cruz Biotechnology; sc-48385), anti-NPM1 (Millipore; B0556), anti-FBL (Santa Cruz Biotechnology; sc-374022), anti-GAPDH (ABclonal; AC033), anti-H3 (Cell Signaling Technology; 4499S), anti-H3K36me2 (Cell Signaling Technology; 2901S), Goat anti-Mouse IgG (H+L) Cross-Adsorbed Secondary Antibody, Alexa Fluor 488 (Invitrogen; A-11001), Goat anti-Rabbit IgG (H+L) Cross-Adsorbed Secondary Antibody, Alexa Fluor 594 (Invitrogen; A-11012), anti–monomethyl lysine (Cell Signaling Technology; 14679S), anti–trimethyl lysine motif (Cell Signaling Technology; 14680S), and anti-GFP (Cell Signaling Technology; 55494S).

### Plasmid construction

The complementary DNA (cDNA) sequences encoding REIIBP or MMSET were cloned into the SFB vector (S protein tag, FLAG tag, and streptavidin-binding peptide) for the expression in mammalian cells and generation of stable cell lines. For protein purification, the cDNAs encoding the following proteins or domains were cloned into the pGEX-6P-1 vector to generate GST-tagged fusion constructs: REIIBP, the Y311A mutant of REIIBP; the PWWP domain, PHD domain, SET domain, and two disordered regions of REIIBP; EXOSC7, the K276A mutant of EXOSC7; NIP7, and the K62A mutant of NIP7. Full-length REIIBP and truncated mutants (ΔPHD-1, ΔPHD-2, ΔPWWP, ΔSET, Δdisordered region-1, Δdisordered region-2, and Δdisordered regions-1 + 2) were cloned into the pEGFP-C1 vector for IF assays. For the generation of stable *REIIBP*-knockdown cell lines, DNA with shRNA sequences targeting *REIIBP* (sh-REIIBP-1, sh-REIIBP-2) were cloned into the lentiviral transfer vector pLV-U6-shRNA-CMV-EGRP-T2A-Puro. All constructs were verified by DNA sequencing. Primer sequences are listed in Table S4.

### Cell culture, cell transfection, and viral transduction

HeLa cells were cultured at 37°C with 5% CO_2_ (v/v) in Dulbecco’s modified Eagle’s medium containing 10% fetal bovine serum and supplemented with 1% penicillin–streptomycin. NCI-H929 cells were cultured in RPMI1640 medium containing 10% fetal bovine serum and supplemented with 1% penicillin and streptomycin at 37 °C with 5% CO_2_ (v/v). Plasmid transfection was performed using Lipofectamine 2000 reagent (Invitrogen; 11668019) following the manufacturer’s instructions. NCI-H929 cells were transduced with lentiviral particles at a multiplicity of infection of 40 in the presence of 10 μg/ml polybrene.

### IF staining

For adherent HeLa cells, cells were seeded on glass coverslips and cultured until ∼80% confluency, then washed twice with PBS. For suspension of NCI-H929 cells, cells were harvested, washed twice with PBS, spotted onto glass slides, and allowed to air-dry briefly at room temperature. Cells were subsequently incubated in CSK buffer (10 mM Hepes [pH 7.0], 100 mM NaCl, 300 mM sucrose, 3 mM MgCl_2_, and 0.7% [v/v] Triton X-100) for 3 min at room temperature for preextraction. Cells were then fixed with 4% paraformaldehyde for 20 min at room temperature, followed by 3 times wash with PBS. After permeabilization with 0.5% [v/v] Triton X-100 in PBS for 10 min, cells were blocked with 2% bovine serum albumin for 1 h at room temperature. Primary antibody incubation was carried out overnight at 4 °C, followed by fluorescent secondary antibody incubation for 1 h at room temperature. Cells were washed 3 times with PBS for 5 min, and glass slides and coverslips were mounted using 4′,6-diamidino-2-phenylindole Fluoromount-G antifade mounting medium. All the fluorescence images were acquired using Zeiss LSM 900 with Airyscan and a 63×/1.4 oil immersion objective and processed using Zen software (Carl Zeiss Microscopy GmbH). Contrast and brightness settings were uniformly applied across experimental groups.

### SIM procedure

SIM images were acquired using multimodality SIM imaging system (NanoInsights-Tech Co, Ltd) equipped with a 100 × 1.49 numerical aperture oil immersion objective and a photometrics Kinetix camera. The laser sources at 488 nm, 561 nm, and 405 nm wavelengths were used. The SIM images were taken using single-slice mode with 50 mW laser power and 30 ms exposure time. Images were then reconstructed and processed using the SIM Imaging Analyzer software (NanoInsights-Tech), which was used for detailed visualization of subcellular structures with enhanced resolution and clarity. Colocalization analyses of fluorescence signals were conducted using ImageJ (National Institutes of Health) software.

### Subcellular fractionation

For each experiment, 5 × 10^7^ cells were used. Cells were harvested and washed twice with PBS. From this point onward, all experimental manipulations were performed on ice or at 4 °C. All cells were resuspended in 10 ml of PBS, and 100 μl cell suspension was collected for the whole cell extract (WCE) sample. The remaining cell suspension was spun at 220*g* at 4 °C for 5 min and then discarded the supernatant. The cell pellets were resuspended in 5 ml of cytoplasmic extract buffer (10 mM Hepes, 60 mM KCl, 1 mM EDTA, 0.075% [v/v] NP-40, 1 mM DTT, and 1 mM PMSF, pH 7.6) and lysed at 4 °C for 30 min and checked under a microscope to ensure the majority of cells were disrupted with remaining intact nuclei. Samples were centrifuged at 220*g* at 4 °C for 5 min. Supernatants were collected and used as the cytoplasmic fractions. The remaining pellet was resuspended in 3 ml of buffer S1 (250 mM sucrose, 10 mM MgCl_2_, and EDTA-free protease inhibitors) and layered over 3 ml of buffer S2 (350 mM sucrose, 0.5 mM MgCl_2_, and EDTA-free protease inhibitors) in a new 15 ml conical tube and spun at 1430*g* for 5 min at 4 °C. The supernatant was removed, and the remaining pellet, containing intact nuclei, was resuspended in 3 ml of S2. The nuclei were sonicated 2 × 60 s at 30% amplitude, 1 s on, 1 s off with a 30 s rest between sonications. The sonicated nuclei in S2 were layered over 3 ml of buffer S3 (880 mM sucrose, 0.5 mM MgCl_2_, and EDTA-free protease inhibitors) in a new 15 ml conical tube, avoiding mixing of the layers. After centrifugation at 3000*g* for 10 min at 4 °C, a 1 ml aliquot of the top-most supernatant was saved as a nucleoplasmic (NP) extract. The remaining supernatant was discarded, and the pelleted nucleoli were resuspended in 500 μl buffer S2 and transferred to a 1.5 ml conical tube. Both the NP extract and resuspended nucleoli were spun at 2800*g* for 5 min. The 250 μl supernatant from the NP extract was transferred to a new tube and saved as the NP sample. The supernatant from the nucleoli was removed, and the nucleoli were resuspended in 250 μl S2 as the NO sample. Benzonase (2 μl; 250 U/μl) (Sigma; E1014) was added to the NP and NO samples and incubated at 4 °C for 30 s to digest nucleic acids and improve solubility of chromatin-bound and NO proteins. The WCE sample was spun at 250*g* for 5 min at 4 °C, the supernatant was removed, and the cell pellet was resuspended in 500 μl 1×extraction buffer (50 mM Tris–HCl [pH 7.5], 500 mM NaCl, 1% [v/v] NP-40, 0.05% sodium dodecyl sulfate, 0.05% sodium deoxycholate, and EDTA-free protease inhibitors). After incubation with benzonase, 250 μl of 2× extraction buffer (100 mM Tris–HCl [pH 7.5], 1000 mM NaCl, 2% [v/v] NP-40, 0.1% sodium dodecyl sulfate, 0.1% sodium deoxycholate, and 2× EDTA-free protease inhibitors) was added to the NP and the NO samples. The WCE, NP, and NO samples were sonicated 2 × 30 s at 15% amplitude, 1 s on, 1 s off, and subsequently spun at 21,000*g* for 10 min at 4 °C. The supernatants were transferred to new tubes as the final WCE, NP, and NO fractions.

### Western blotting

Cells were harvested in PBS and centrifuged at 12,000 rpm for 1 min. The pellet was lysed with NETN100 buffer (0.5% [v/v] NP-40, 50 mM Tris–HCl [pH 8.0], 2 mM EDTA, and 100 mM NaCl) containing protease inhibitor cocktail for 30 min. After loading buffer (2% sodium dodecyl sulfate, 100 mM DTT, 100 mM Tris–HCl [pH 6.8], 10% glycerol, 5% 2-mercaptoethanol, and 0.0025% bromophenol blue) was added, the samples were boiled at 95 °C for 5 min. Proteins were separated on SDS-PAGE and were transferred onto nitrocellulose membranes. The nitrocellulose membranes were incubated in blocking buffer (5% bovine serum albumin in TBST buffer (50 mM Tris–HCl, 150 mM NaCl, and 0.1% [v/v] Tween-20) for 1 h. The primary antibody was incubated overnight at 4 °C, and the secondary antibody was incubated for 1 h at room temperature. Blot signals were captured by ChemiDoc XRS+ System using SuperSignal West Pico PLUS kit (Thermo Fisher Scientific; 34580).

### Histone extraction

About 5 × 10^5^ to 1 × 10^6^ cells were collected and washed twice with PBS. Then, the sample was resuspended in 200 μl of high-salt NETN buffer (20 mM Tris [pH 8.0], 500 mM NaCl, 0.5% [v/v] NP-40, and 1 mM EDTA) containing protease inhibitor cocktail and lysed on the ice for 15 min. After centrifugation at 4 °C for 10 min at 1500*g*, a near-transparent precipitate can be seen at the bottom of the tube. After discarding the supernatant, 100 μl of 0.2 M HCl was added to the precipitate and lysed on the ice for 30 min. After centrifugation at 12,000 rpm at 4 °C for 15 min, the supernatant was transferred to a new centrifuge tube, and 20 μl of 1 M Tris–HCl (pH 8.0) was added to neutralize the acidity. SDS loading buffer was added into the sample and boiled at 95 °C for 5 min used for the following Western blotting experiment.

### PAR-CLIP assay

After a 16-h treatment with 0.1 mM 4-thiouridine, HeLa cells stably expressing SFB-tagged REIIBP were washed once with PBS in the plates. The PBS was then completely removed, and the plates were placed on a tray filled with ice to maintain a cold temperature. The cells were subsequently irradiated with 365 nm UV light, delivering a total energy of 0.4 J/cm^2^. Then, the cells were collected and lysed by freshly prepared cold NETN300 lysis buffer (50 mM Tris–HCl [pH 7.5], 300 mM NaCl, 2 mM EDTA, 1% [v/v] NP-40, and 0.5 mM DTT) containing protease inhibitor cocktail and RNase inhibitor. The cell lysates were centrifuged at 12,000 rpm for 15 min at 4 °C, and the supernatant was transferred to a new tube and diluted with NETN0 (50 mM Tris–HCl [pH 7.5], 2 mM EDTA, 1% [v/v] NP-40, and 0.5 mM DTT) to the final concentration as NETN100 (50 mM Tris–HCl [pH 7.5], 100 mM NaCl, 2 mM EDTA, 1% [v/v] NP-40, and 0.5 mM DTT). High-capacity streptavidin resin (Thermo Fisher; 20359) was added into the lysates and incubated for 2 h at 4 °C with rotation. After IP, the resins were washed three times with cold NETN300 wash buffer (50 mM Tris–HCl [pH 7.5], 300 mM NaCl, 0.05% [v/v] NP-40, and 0.5 mM DTT), two times with cold NETN100 wash buffer (50 mM Tris–HCl [pH 7.5], 100 mM NaCl, 0.05% [v/v] NP-40, and 0.5 mM DTT). The interactomes were eluted twice with 500 μl of 2 mg/ml biotin for 15 min at 4 °C. Next, anti-FLAG M2 beads (Millipore; A2220) were added into the elution and incubated another 1.5 h at 4 °C with rotation. After the second pull-down, the beads were washed with cold NETN300 wash buffer three times and cold NETN100 wash buffer twice, respectively. The coprecipitated RNAs were released by proteinase K (Beyotime; ST532) and extracted by TRIzol RNA extraction reagent (Thermo Fisher; 15596018CN) according to the manufacturer's instructions. Purified RNAs were used for RNA-Seq or RT–qPCR.

The quality of RNA samples was examined by Qubit 3.0 Fluorometer (Invitrogen) and Agilent 2100 Bioanalyzer (Applied Biosystems), respectively. RNA-Seq was performed by Geneseed Bio Technology Co, Ltd. The trimmed RNA-Seq reads were aligned to the human genome assembly T2T-CHM13v2.0. The total RNA amount purified from SFB-REIIBP and the empty control (SFB-vector) is compared. The relative amount of purified rRNA is calculated based on the percentage of sequencing reads of rRNA in total RNA and the amount of total RNA.

### *In vitro* RNA and GST-protein pull-down assay

Purified N-terminal GST-tagged proteins (3 μg), total RNAs (native RNA/heat-denatured RNA) (3 μg), and Glutathione Sepharose 4B (200 μl) beads (Cytiva; 17075604) were incubated in 500 μl NETN100 buffer with RNase inhibitor (Promega; N2611) at 4 °C for 2 h. Then, the beads were centrifuged and washed with NETN300 buffer three times and NTN100 buffer three times. Then, the supernatant was discarded, and protein-associated RNA was extracted by TRIzol RNA extraction reagent for RT–qPCR.

### RT–qPCR

For RT–qPCR, the PrimerScript First Strand cDNA Synthesis Kit (Takara; 6210A) was used to synthesize cDNA with random hexamer primers or oligo(dT) primers. qPCR was performed with triplicate samplings of retrotranscribed cDNAs on CFX Connect Real-Time PCR Detection System (Bio-Rad; SIA-PCR007) using Hieff SYBR Green Master Mix (YEASEN; 11201ES03). Primers are listed in Table S4. The mean value was calculated by 3 independent experiments. Fold change was calculated by 2^(-ΔΔCt)^ algorithm.

### Northern blotting

Total RNA was purified from HeLa cells (SFB-REIIBP/SFB-vector-expressing) and NCI-H929 cells (*REIIBP*-knockdown/nontargeting control) with TRIzol RNA extraction reagent according to the manufacturer’s protocol. RNA (2 μg) was loaded on the 1.2% denatured agarose gel and separated in MOPS buffer for 3 to 4 h at 4 °C, then transferred to positively charged Amersham Hybond-N+ membranes (Cytiva; RPN203B) in 20× saline sodium citrate (SSC) buffer overnight. Then, the membranes were washed with 2× SSC and air dried at 80 °C for 30 min. After UV crosslinking, the membranes were hybridized respectively with biotin-labeled 5′ETS, ITS1, ITS2, and GAPDH probes in Ambion ULTRAhyb-Oligo buffer (Invitrogen; AM1940) at 42 °C overnight. The membranes were washed with wash buffer (0.2× SSC, 0.1% SDS) for 15 min 3 times at 42 °C. Finally, the bolt signals were detected by ChemiDoc XRS+ System using Chemiluminescent Nucleic Acid Detection Module Kit (Thermo Scientific; 89880). The sequences of biotin-labeled probes are listed in Table S4.

### Expression and purification of recombinant proteins

Expression of GST-tagged recombinant proteins was induced with 0.1 mM IPTG in *Escherichia coli* strain BL21. Then, the bacteria were pelleted and resuspended with 20 ml of NETN100 buffer (20 mM Tris–HCl [pH 8.0], 100 mM NaCl, 1 mM EDTA, and 0.1% [v/v] Triton X-100) containing 2 mM PMSF and 1 mM DTT. Next, the cell lysates were sonicated for 10 min at 35% amplitude, 10 s on, 10 s off on the ice. After centrifugation at 10,000 rpm for 20 min at 4 °C, the supernatant was collected and incubated with glutathione beads at 4 °C for 2 h with rotation. After NETN300 wash, the beads were eluted with 10 mM l-glutathione diluted in NETN100. After dialysis, proteins in PBS were stored at −80 °C.

### IP–MS

HeLa cells stably expressing SFB-REIIBP or SFB-vector were lysed with radioimmunoprecipitation assay buffer (Beyotime; P0013B), respectively. The lysates were centrifuged at 13,000 rpm for 30 min at 4 °C. Then the supernatant was incubated with high-capacity streptavidin resin at 4 °C for 2 h. After washing the resin 3 times with NETN100 buffer, bound proteins were eluted using 2 mg/ml biotin. A second round of pull-down was performed using anti-FLAG M2 affinity gel (Millipore; A2220). After an additional 2-h incubation and 3 washes, the eluted proteins were separated by SDS-PAGE and stained with Coomassie Blue G-250. Gel bands were excised, reduced, and alkylated in 50 mM ammonium bicarbonate at 37 °C overnight, followed by tryptic digestion. Peptides were extracted twice with 0.1% formic acid in 50% acetonitrile, concentrated by SpeedVac, and analyzed by LC–MS/MS using a Thermo Vanquish Neo nano-HPLC system interfaced with a Thermo Q Exactive HF-X mass spectrometer. The mass spectrometer was operated in a data-dependent acquisition mode, using Xcalibur 4.1 software, performing full scans in the Orbitrap (400–1800 *m/z*, 60,000 resolution), followed by 20 data-dependent MS/MS scans at 30% normalized collision energy. The resulting MS data were analyzed using Thermo Xcalibur Qual Browser and Proteome Discoverer, with searches against the *Homo sapiens* proteome database (UniProtKB, UP000005640). Sequest search parameters included a 10 ppm precursor mass tolerance, a 0.02 Da fragment ion tolerance, and up to two internal cleavage sites. Peptides were filtered at a 1% false discovery rate.

### Protein methylation MS

NO proteins were enriched using the subcellular fractionation method described previously. Protein concentration was determined *via* a bicinchoninic acid assay (Thermo Scientific; 23225). Then, a total of 10 μg of protein was reduced with 10 mM DTT at 55 °C for 45 min, followed by alkylation with 50 mM iodoacetamide at room temperature in the dark for 30 min. Purification was carried out using SP3 technology, as described by Hughes *et al*. (44). The purified proteins were digested overnight at 37 °C with trypsin (Promega; V5280) at a 1:100 enzyme-to-protein ratio (w/w). The resulting peptides were lyophilized in preparation for LC–MS/MS analysis.

For LC–MS/MS, lyophilized peptides were resuspended in 20 μl of 0.1% formic acid, and 2 μl aliquots were injected into the nanoElute UHPLC System (Bruker). After gradient elution, peptides were analyzed using a TIMS quadrupole time-of-flight timsTOF Pro 2 instrument (Bruker Daltonics) with a CaptiveSpray nanoelectrospray source. Data were collected in data-dependent acquisition-parallel accumulation–serial fragmentation mode, with an *m/z* range of 300 to 1500 and a 1/K0 range of 0.75 to 1.3, using a ramp time of 166 ms.

Label-free quantification was performed with MSFragger *via* FragPipe v20.0, MSFragger 3.8, and Philosopher 5.0.0, employing the label-free quantification-match-between-runs workflow (45, 46). Search parameters matched those used in the protein identification step, with IonQuant settings, including a mass tolerance of 20 ppm, a retention time tolerance of 3 min, and an ion mobility tolerance of 0.05. Fixed modifications included cysteine alkylation, whereas methionine oxidation and lysine monomethylation, dimethylation, and trimethylation were set as variable modifications. Normalization was enabled, and a minimum isotope count was set to 2. Peptides were filtered at a 1% false discovery rate.

### *In vitro* methylation assay

Recombinant GST-REIIBP (wildtype or the catalytically inactive Y311A mutant; 0.5 μg) was incubated with purified recombinant EXOSC7, EXOSC7(K276A), NIP7, or NIP7(K62A) (3 μg each) in methylation buffer (50 mM Tris [pH  8.0], 20 mM KCl, 5 mM MgCl_2_, 10% glycerol, and 1 mM DTT) containing 80 μM *S*-adenosylmethionine (New England Biolabs; B9003S) in a total reaction volume of 50 μl. Reactions were performed at 30 °C for 1 h and terminated by boiling with SDS loading buffer. Reaction mixtures were then resolved by SDS-PAGE, and protein methylation levels were analyzed by immunoblotting using antibodies specific for monomethylated lysine or trimethylated lysine.

### Polysome profiling

Cells were treated with cycloheximide (100 mg/ml) for 15 min, and then 7 × 10^7^ cells were harvested and lysed in lysis buffer (20 mM Tris–HCl [pH 7.4], 15 mM MgCl_2_, 200 mM KCl, and 1% [v/v] Triton X-100) supplemented with 40 U/ml RNasin Ribonuclease Inhibitor (Promega; N2611), complete ULTRA protease inhibitor (Roche; 4693132001), 100 mg/ml cycloheximide, and 1 mM DTT. Cell lysates were centrifuged at 13,000 rpm at 4 °C for 10 min, and the supernatant was loaded onto 10% to 45% sucrose gradients followed by ultracentrifugation with SW41 rotor (Beckman) at 36,000 rpm at 4 °C for 3 h. Absorbance at 260 nm was recorded using a BioComp Piston Gradient Fractionator equipped with a Bio-Rad Econo UV Monitor. Polysome-to-monosome ratios were calculated by comparing the areas under the monosome and polysome peaks.

### Statistical analysis

All data were analyzed with GraphPad Prism 10.0 (GraphPad Software, Inc) software. The data were presented as mean ± SD. Statistical significance between the two groups was subjected to the two-tailed Student’s *t* test. *p* < 0.05 was considered statistically significant.

## Data availability

All the relevant data associated with the current study can be found in the main text or the supporting information. RNA-Seq data have been deposited in the Gene Expression Omnibus datasets under the Gene Expression Omnibus accession number GSE294414. The MS proteomics data in this study have been deposited to the ProteomeXchange Consortium with the identifiers PXD062854 and PXD062856.

## Supporting information

This article contains supporting information.

## Conflict of interest

The authors declare that they have no conflicts of interest with the contents of this article.
