# Supplementary material for: REIIBP methylates nucleolar proteins and regulates pre-rRNA processing
Source: J Biol Chem. 2025 Aug 16;301(10):110609. doi: 10.1016/j.jbc.2025.110609 (PMC12466271; doi:10.1016/j.jbc.2025.110609)
Supplement: Supporting Information [file mmc1.pdf]

Supporting information for

REIIBP methylates nucleolar proteins and regulates pre-rRNA processing

Qianqian Yang[1,2,3,4] and Xiaochun Yu[2,3,4]*

This file includes:

Figures S1 to S5

Tables [S1-S4] legends

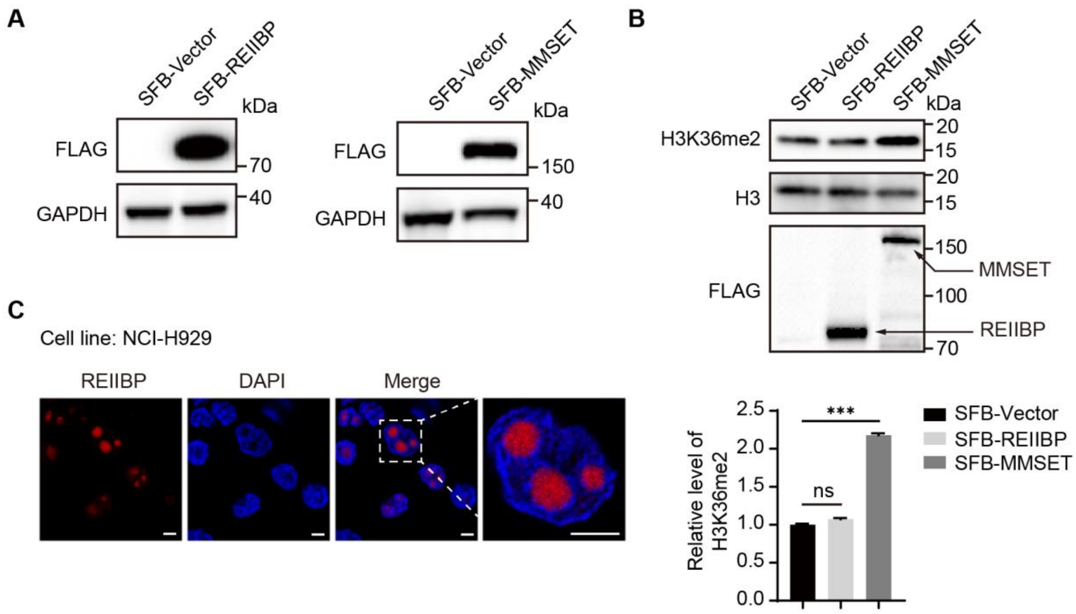

Figure S1: REIIBP does not affect the global level of H3K36me2. (A) SFB-tagged REIIBP or MMSET are stably expressed in HeLa cells. WB assays were performed with anti-FLAG antibody. GAPDH was used as a protein loading control. **(B)** REIIBP does not affect global H3K36me2 levels. The global H3K36me2 levels were examined by WB assays with the indicated antibodies. SFB-MMSET was used as a positive control. The relative levels of H3K36me2 (normalized to H3) are displayed in the lower panel of (C). ***p < 0.001, ns, not significant. **(C)** REIIBP localizes at nucleolus in NCI-H929 cells. Endogenous REIIBP was examined by IF with anti-REIIBP antibody. Image bar: 5 μm.

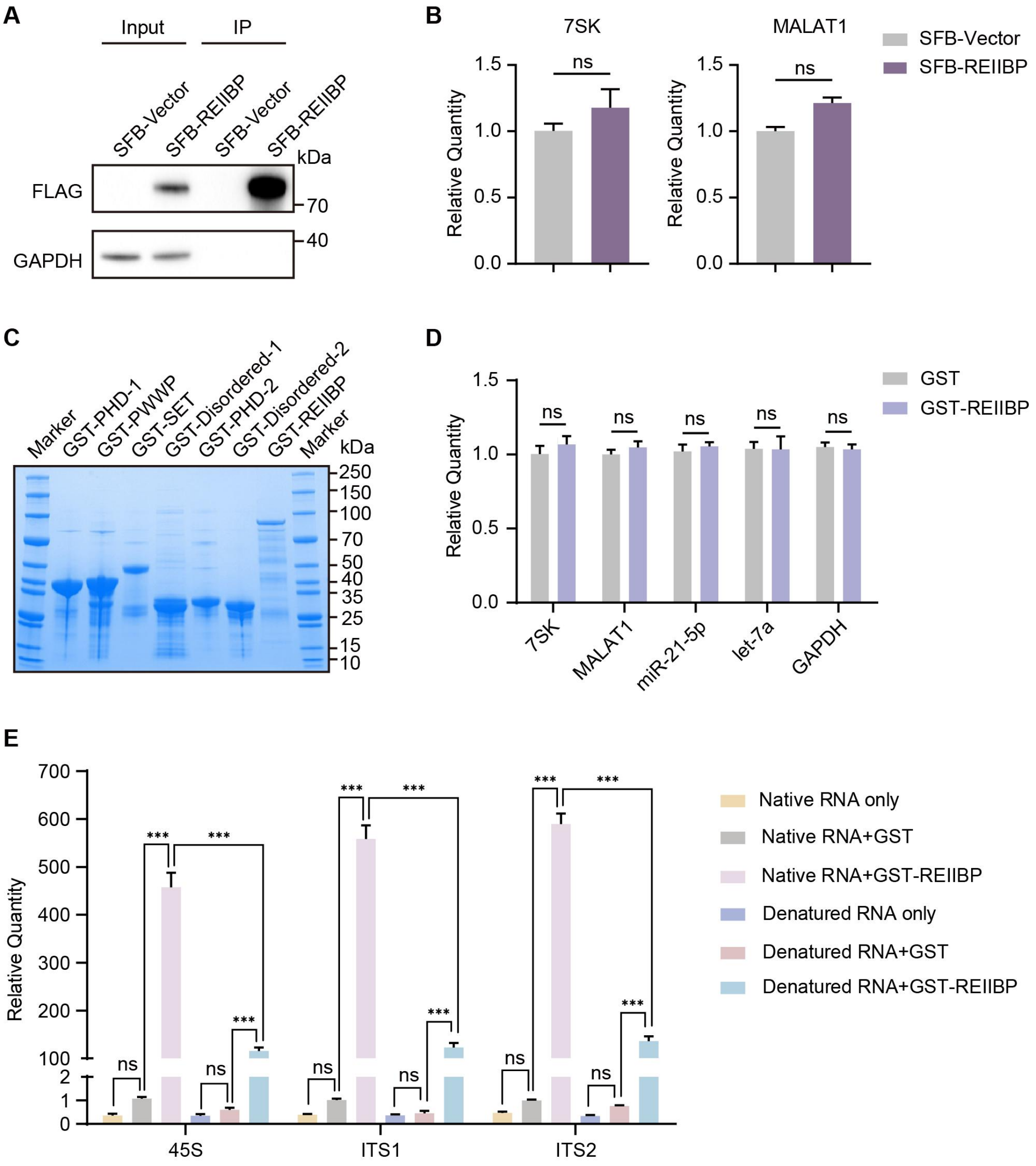

Figure S2: REIIBP does not bind to other RNA species. (A) WB assay detected the immunoprecipitation efficiency of SFB-REIIBP in the PAR-CLIP assay. **(B)** REIIBP does not bind to 7SK RNA or MALAT1 RNA. Immunoprecipitation was performed in HeLa cells stably expressing SFB-REIIBP or only SFB-vector, 7SK and MALAT1 were examined by RT-qPCR. ns, not significant. **(C)** Purification of recombinant full-length REIIBP and truncated REIIBP proteins. GST-tagged proteins were analyzed by electrophoresis and stained with Coomassie Brilliant Blue. **(D)** Recombinant REIIBP protein does not bind LncRNA, miRNA, snRNA or mRNA *in vitro*. GST or GST-REIIBP proteins were incubated with total RNA extracted from

HeLa cells. RT-qPCR was performed to examine REIIBP-associated RNAs using primers targeted on 7SK, MALAT1, miR-21-5p, let-7a and GAPDH. ns, not significant. (E) Pre-rRNA secondary structure influences its binding with REIIBP. GST or GST-REIIBP proteins were incubated with native RNA or heat-denatured RNA extracted from HeLa cells. RT-qPCR was performed to examine REIIBP-associated pre-rRNAs using primers targeted on 5'ETS, ITS1 and ITS2 regions of pre-rRNA. ***$P < 0.001$, ns, not significant.

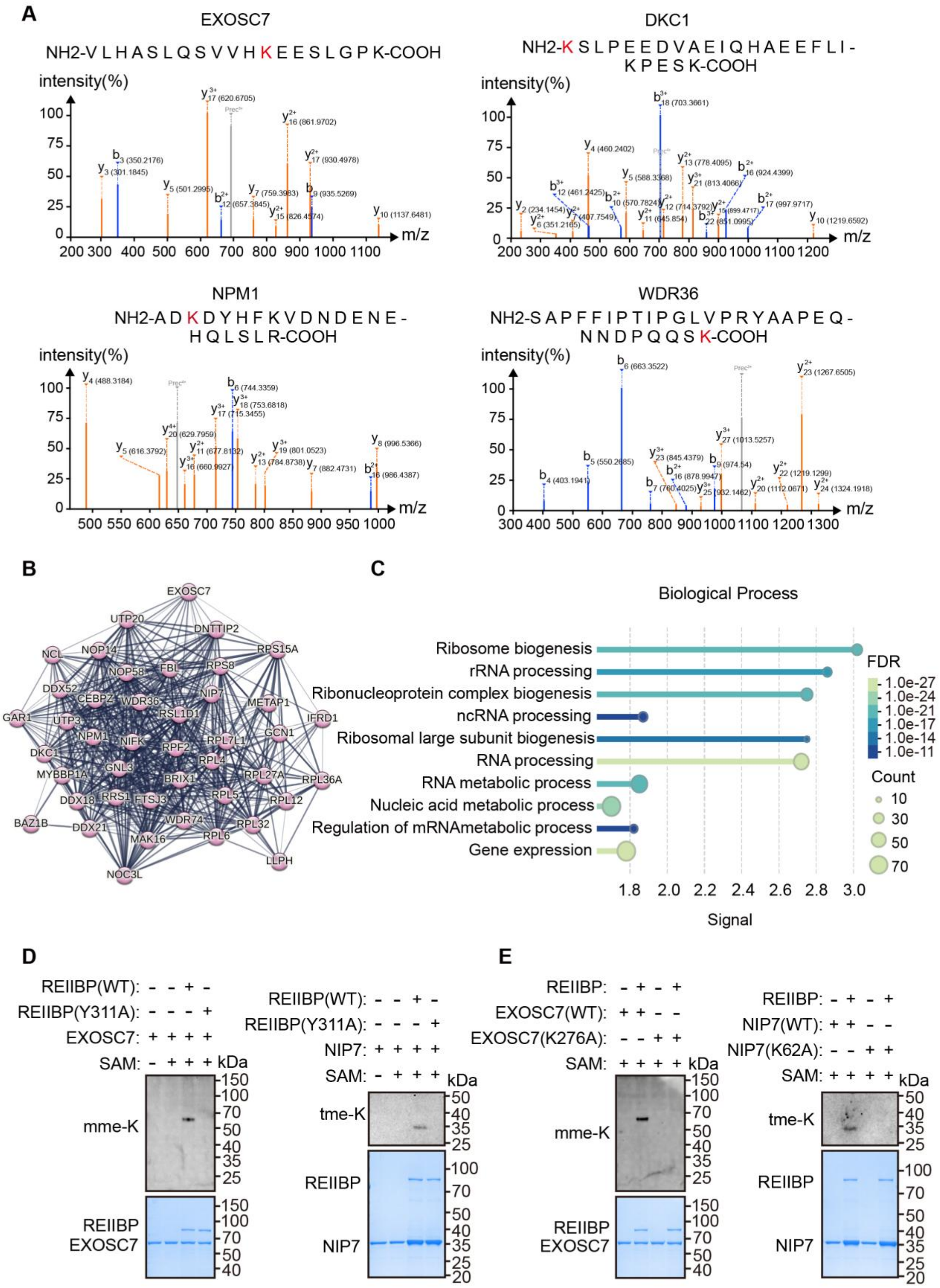

Figure S3: REIIBP methylates pre-rRNA processing proteins. (A) MS/MS spectra of four representative peptides with elevated lysine methylation levels. The m/z values of experimentally observed y-ions and b-ions matched with the theoretical fragmentation patterns. The lysine residues marked in red within the peptide

sequences were identified as the specific site of methylation modification. **(B)** Network presentation of physical connections between proteins in the largest cluster from the methylation upregulated substrates of REIIBP. **(C)** GO analysis shows that the candidate substrates of REIIBP are primarily enriched in ribosome biogenesis and rRNA processing pathway. **(D-E)** REIIBP methylates K276 of EXOSC7 and K62 of NIP7. *In vitro* methylation assays were performed by incubating GST-REIIBP (wild-type or catalytically inactive mutant Y311A) with EXOSC7 or NIP7. Methylation was detected by WB assays using antibodies against mono-methyl lysine (mme-K) or tri-methyl lysine motif (tme-K) **(D)**. *In vitro* methylation assays were also performed by incubating GST-REIIBP with EXOSC7(K276A) or NIP7(K62A) **(E)**.

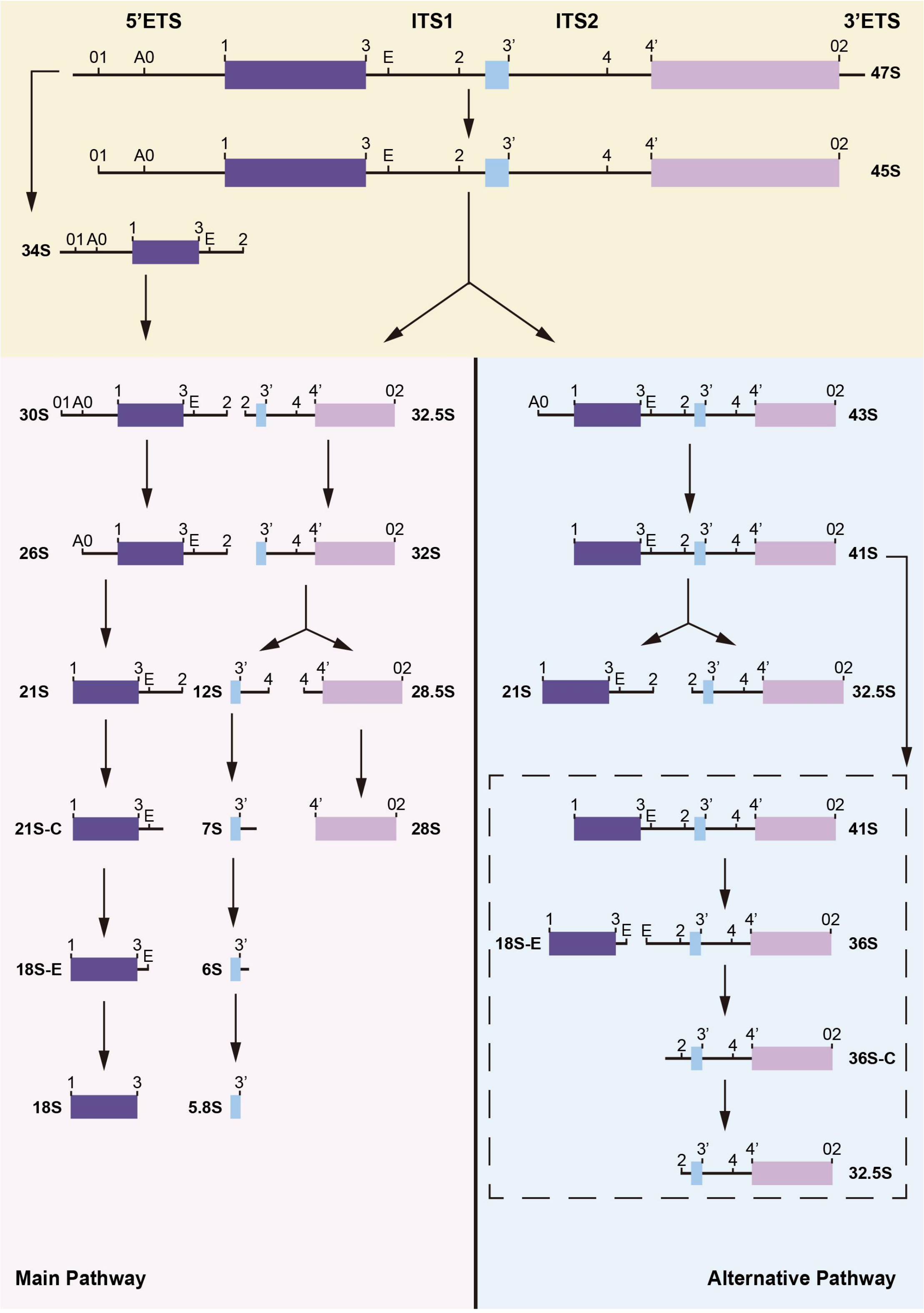

Figure S4: Schematic representation of pre-rRNA processing pathways in human cells. The 18S, 5.8S, and 28S rRNAs are processed from 47S pre-rRNA transcript synthesized by RNA polymerase I at NO rDNA clusters. The 5S rRNA (not shown) is independently transcribed by RNA polymerase III from chromosome 1. The 47S

pre-rRNA contains mature rRNA sequences interspersed with 5'ETS, ITS1, ITS2 and 3'ETS, which are systematically removed through sequential cleavage events. The yellow module highlights the early stages of pre-rRNA processing, including cleavage at sites 01 and 02 to generate 45S pre-rRNA or cleavage at site 2 before site 01 to generate 34S pre-rRNA. The cleavage of the 45S pre-rRNA can either start in the 5'ETS (site A0) or in the ITS1 (site 2), defining two pathways: the main processing pathway (pink module) and the alternative processing pathway (blue module), with minor routes indicated in the dashed box. Exonuclease cleavage sites are marked on each pre-rRNAs.

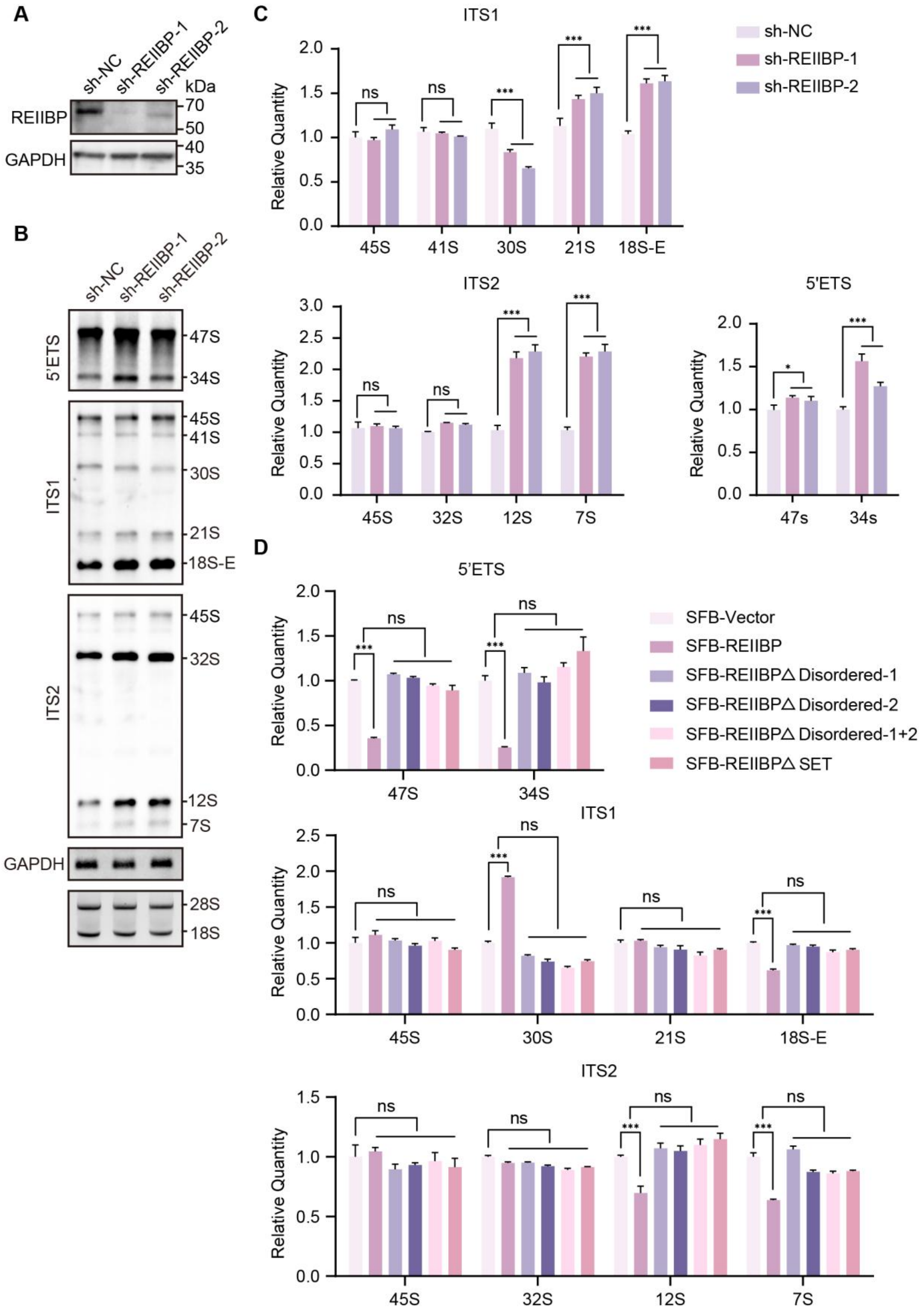

Figure S5: Regulation of pre-rRNA processing by REIIBP depends on its disordered regions and SET domain. (A) REIIBP is knocked down by shRNA in NCI-H929 cells. The stable knockdown of REIIBP in NCI-H929 was examined by WB assay using the indicated antibodies. shRNA sequences are provided in Table S4.

(B-C) REIIBP knockdown affects pre-rRNA processing. NB analyses were performed to examine pre-rRNA intermediates in NCI-H929 cells stably knockdown endogenous REIIBP with indicated probes shown in figure 6A **(B)**. The relative levels of 47S, 45S, 41S, 30S, 21S, 18S-E, 32S, 12S and 7S pre-rRNAs are measured. Values are means ± SD of three independent assays. ***$p < 0.001$, ns, not significant **(C)**. **(D)** Quantitative analysis of the Northern blots in figure 6C. Values are means ± SD of three independent assays. ***p < 0.001, ns, not significant.

Table S1. List of proteins identified by IP-MS in HeLa cells stably expressing REIIBP.

Table S2. GO enrichment analysis (biological process, cellular component, and molecular function) and KEGG pathway enrichment analysis of proteins upregulated in IP-MS experiments.

Table S3. List of proteins with elevated lysine methylation levels in the presence of REIIBP identified by methylation mass spectrometry.

Table S4. Sequences of oligonucleotide primers for PCR and qPCR, probes for NB assays, and shRNA.
